# C-Type Natriuretic Peptide Pre-Treatment Improves Maturation Rate of Goat Oocytes by Maintaining Transzonal Projections, Spindle Morphology, and Mitochondrial Function

**DOI:** 10.3390/ani13243880

**Published:** 2023-12-16

**Authors:** Rui Xu, Menghao Pan, Lu Yin, Yiqian Zhang, Yaju Tang, Sihai Lu, Yan Gao, Qiang Wei, Bin Han, Baohua Ma

**Affiliations:** 1 Key Laboratory of Animal Biotechnology, Ministry of Agriculture, College of Veterinary Medicine, Northwest A&F University, Yangling 712100, China; xr1127419533@163.com (R.X.); panmenghao@nwafu.edu.cn (M.P.); yinlu19990428@163.com (L.Y.); zyq1027219294@163.com (Y.Z.); weiq@nwsuaf.edu.cn (Q.W.); 2 Yulin Animal Husbandry and Veterinary Service Center, Yulin 719000, China

**Keywords:** goat oocyte, C-type natriuretic peptide, transzonal projections, mitochondria, spindle

## Abstract

**Simple Summary:**

C-type natriuretic peptide (CNP) is a member of a family of natriuretic peptides that are expressed in mammals and have an important role in the recovery of meiosis. The aim of this research was to examine the effect of CNP on meiosis of goat oocytes. The results showed that pre-treatment of a goat cumulus–oocytes complex with CNP before maturation culture promoted the maturation of goat oocytes. Experiments revealed that CNP can control transzonal projection (TZPs) in order to maintain communication between cumulus cells and oocytes, and it can also control the assembly of the oocyte meiosis spindle apparatus. Further analysis showed that pre-treatment of goat cumulus–oocytes complex with CNP had an improved antioxidant capacity and increased mitochondrial activity. In conclusion, these findings shed new light on the potential mechanism of CNP treatment to enhance oocyte quality before fertilization.

**Abstract:**

C-type natriuretic peptide (CNP) is a peptide molecule naturally found in follicles and can be used to extend meiotic resumption and enhance the potential for oocytes to develop. However, the mechanism by which CNP improves goat oocyte quality remains unclear. In this study, cumulus–oocyte complexes (COCs) from goats were pre-treated with CNP prior to IVM, and the results showed that pre-treatment with CNP enhanced goat oocyte maturation. First, we discovered that CNP maintained communication between cumulus cells and oocytes by regulating the transzonal projections (TZPs). We then found that CNP treatment reduced abnormal spindle formation and increased the expression of genes associated with spindle assembly and the spindle assembly checkpoint. Moreover, further analysis showed that oocytes exhibited better antioxidant ability in the CNP treatment group, which mainly manifested in higher glutathione (GSH) and lower reactive oxygen species (ROS) concentrations. Enhanced mitochondrial activity was signified via the augmented expression of mitochondrial oxidative metabolism and fusion and fission-related genes, thus diminishing the apoptosis of the oocytes. Overall, these results provide novel insights into the potential mechanism by which CNP treatment before IVM can improve oocyte quality.

## 1. Introduction

Oocyte in vitro maturation (IVM) has been widely used as an assisted reproductive technology, especially in the livestock breeding industry. However, conventional IVM tends to provide poor oocyte maturation quality, which is due to oocytes isolated from antral follicles exhibiting asynchronous nuclear and cytoplasmic maturation [1,2]. In recent years, researchers have found that a small-molecule polypeptide naturally present in follicular fluid, known as C-type natriuretic peptide (CNP), acts as a meiotic inhibitor, delaying the spontaneous recovery of oocyte meiosis [3]. CNP is synthesized and secreted by follicle mural granulosa cells, and then, combining with and activating its receptor, natriuretic peptide receptor 2 (NPR2), which is located on cumulus cells, promotes the production of cyclic guanosine monophosphate (cGMP) in cumulus cells. cGMP is transferred into the oocyte via transzonal projections (TZPs) and gap junctions (Gj) to inhibit phosphodiesterase 3A (PDE3A) activity, causing cyclic adenosine monophosphate (cAMP) degradation to slow down and, in turn, maintaining a high cAMP level and meiotic arrest [3,4]. Several species have demonstrated an enhancement in oocyte maturation quality and blastocyst development through the implementation of CNP pre-treatment prior to IVM [5,6,7,8].

The maturation of oocytes is a multifaceted and meticulously controlled process that entails various biological events, including interactions between somatic cells and oocytes, the resumption of meiosis, cytoskeletal dynamics, and cytoplasmic reorganization [9]. TZPs are thin, actin-rich cytoplasmic projections that extend from the cumulus cells and through the zona pellucida, contacting the oocyte membrane through the adherens junctions and gap junctions [10]. It is now well established that somatic cells, via TZPs and Gj, emit metabolic and regulatory signals to oocytes, which are necessary for oocyte development and maturation. Meanwhile, oocyte-secreted factors (OSFs) are transported to granulosa and cumulus cells via these channels to regulate their proliferation and follicular growth rate [11,12].

Meiosis is one of the decisive events in oocyte maturation; however, prolonged arrest in dictyate appears to predispose oocytes to missegregate chromosomes and generate aneuploidies [13]. During metaphase I (MI), the proper attachment of homologous chromosomes to the kinetochores of microtubules is crucial for accurate chromosome segregation during anaphase. Inaccurate attachment between the kinetochores and spindle microtubules can result in unstable chromosome segregation, ultimately leading to aneuploidy [14]. The spindle assembly checkpoint (SAC) is a surveillance mechanism that promotes accurate chromosome segregation, involving several molecules such as the Mad and Bub families [15].

In addition to correct chromosome segregation, complex structural and biochemical changes in the cytoplasm are necessary for oocytes to undergo normal fertilization and sustain embryonic development [16]. Mitochondria, being one of the crucial organelles in the cytoplasm, are predominantly inherited from oocytes during zygote formation and the initial stages of embryonic growth [17]. The adequate distribution of mitochondria, the ability of oxidative phosphorylation in generating ATP, the copy number or transcription level of mitochondrial DNA, and the functionality of mitochondria are essential for oocyte maturation, fertilization and embryonic development. Moreover, the state of the mitochondria serves as a parameter in gauging the development of the cytoplasm [18].

In recent years, there have been reports both at home and abroad about the effects of the CNP on oocyte maturation in mammals, but few studies have focused on the specific mechanism by which the CNP regulates goat oocyte maturation. Therefore, it is of great significance to delve into the regulatory mechanism of CNP in oocyte maturation to reveal the reproductive laws of mammals and improve the economic benefits of animal husbandry.

## 2. Methods

### 2.1. IACUC Statement

The collection and use of goat ovaries in this study were subject to review and approval by the Institutional Animal Care and Use Committee of the College of Veterinary Medicine, Northwest A&F University (IACUC number 2021031).

### 2.2. Antibodies and Chemicals

The C-type natriuretic peptide was obtained from ApexBio (Boston, MA, USA, Cat# B5441), the Goat cGMP Assay kit and Goat cAMP Assay kit were obtained from MEIMIAN (Yancheng, Jiangsu, China, Cat# MM-75100O2, MM-1274O2), phalloidin-FITC (Cat# SML0006) and anti-α-tubulin-FITC antibody (Cat# P5282)were obtained from Sigma (St. Louis, MO, USA); Medium 199 was obtained from Gibco (Carlsbad, CA, USA, Cat# 11043023), The mitochondrial membrane potential assay kit (Cat# C2003S), Mito-Tracker Red CMXRos (Cat# C1035), Reactive Oxygen Species Assay Kit (Cat# S0033S), and GSH and GSSG Assay Kit (Cat# S0053) were obtained from Beyotime (Shanghai, China). Unless otherwise indicated, all other chemicals were purchased from Sigma.

### 2.3. COCs Isolation and In Vitro Maturation

The ovaries were obtained from a local slaughterhouse, placed in a sterile physiological saline solution including 75 μg/mL penicillin and 50 μg/mL streptomycin, and subsequently transferred to the laboratory in a Thermos bottle within a time frame of 1–4 h. Upon transportation to the laboratory, the goat ovary underwent a thorough cleaning process involving the removal of surrounding tissues and washing with normal saline. Subsequently, the goat COCs were isolated by needling 2–6 mm follicles of the ovarian surface. COCs exhibiting uniform ooplasm and a cumulus cell layer exceeding three were purposefully selected for the subsequent analysis. Depending on the design of the experiment, the COCs were cultured in a CNP pre-treatment medium plus IVM or IVM. In each group, a total of 30–50 germinal vesicle (GV) COCs were selected and cultured in drops covered with mineral oil at different times at 38.5 °C in a humidified atmosphere of 5% CO_2_.

CNP pre-treatment medium: Basic TCM-199 supplemented with 50 nM CNP.

IVM medium: Basic TCM-199 supplemented with 1 mM Na-pyruvate, 2.5 mM glutamine, 50×NEAA, 50× ITS, 10% FBS, 1 μg/mL Estradiol, 0.075 IU/mL HMG, 10 ng/mL EGF, 75 μg/mL penicillin, and 50 μg/mL streptomycin. The culture time was set according to the different experiment designs. The COCs were cultured in vitro for 14 h (reaching the MI stage) or 24 h (reaching the MII stage).

For GV-stage oocytes, the oocyte nucleus is large, the chromatin is highly loose, and the nuclear membrane is intact. The oocytes’ nuclei were visualized using DAPI staining in order to accurately distinguish GV-stage oocytes. For the MII-stage oocytes, we observed the oocytes using the first polar body extrusion under a microscope.

### 2.4. Immunofluorescence Staining

The oocytes were fixed with 4% paraformaldehyde for 30 min, followed by permeabilization with 1% Triton X-100 in PBS (phosphate-buffered saline) for 1 h. They were further blocked with 1% BSA-supplemented PBS for 1 h at room temperature (RT). Next, the oocytes were incubated with Phalloidin-FITC (1:1000) or α-tubulin-FITC antibody (1:1000) at RT for 3 h, then washed three times using PBST (PBS with 0.1% Tween 20). Subsequently, the oocytes were incubated with Hoechst 33342 at RT for 15 min. Following the staining process, the samples were transferred onto glass slides and subsequently examined using confocal microscopes (Nikon Eclipse Ti, Tokyo, Japan; Leica TCS SP8, Wetzlar, Germany).

We used the counting function of ImageJ to count the number of TZPs in the zona pellucida to quantify the TZPs. The fluorescence intensity of F-actin in the zona pellucida was calculated using ImageJ to quantify the F-actin.

A normal spindle is identified when the oocyte presents a columnar spindle shape with the chromosomes located at the center of the equatorial plate. However, an abnormal spindle is identified via features such as non-aggregation, multipolarity, and an irregular shape.

### 2.5. Measurement of cGMP and cAMP Levels in Oocytes

A total of 100 COCs in each group were collected and cultured. After culturing, the cumulus cells were removed to obtain oocytes. In accordance with the guidelines provided by the manufacturer, the Goat cGMP Assay kit and Goat cAMP Assay kit were used to quantify the concentrations of cGMP and cAMP.

### 2.6. Measurement of Intra-Oocyte Reactive Oxygen Species (ROS) and Glutathione (GSH) Levels

The MII-stage oocytes’ ROS levels were measured by staining them with DCFH-DA. Briefly, the denuded oocytes were subjected to incubation with 25 μg/mL DCFH-DA for a period of 15 min at a temperature of 37 °C in an environment devoid of light. Following this, the oocytes were washed thrice in PBS at a temperature of 37 °C and then immediately transferred onto glass slides and scrutinized using a fluorescence microscope. ImageJ V1.8.0 software was used to compare the green fluorescence intensity in each group.

The oocytes’ GSH content was measured using a GSH and GSSG assay kit as per the manufacturer’s instructions. The content was measured on a microplate reader (BioTek microplate reader). The GSH content was expressed as pmol/oocyte, and the GSH content of the test samples was calculated as Total Glutathione − GSSG × 2. The GSH content was measured according to the manufacturer’s instructions.

### 2.7. Oocyte MitoTracker Staining

The oocytes were incubated with 100 nM MitoTracker Red for 30 min at 37 °C, transferred into 37 °C 4% paraformaldehyde, fixed for 15–30 min, and washed in 37 °C PBS three times. All of the above steps were performed in the dark. Then, the oocytes were expeditiously placed onto glass slides and using confocal microscopes. The fluorescence intensity and the fluorescence distribution in the oocytes were compared using ImageJ V1.8.0 software. The fluorescence intensity reflected the mitochondrial content in the oocytes.

### 2.8. Measurement of Mitochondrial Membrane Potential (MMP)

MII-stage oocytes were subjected to an incubation process in the presence of a 5 μg/mL JC-1 probe for a duration of 30 min at 37 °C and then washed with PBS at 37 °C three times. These two steps were performed in the dark. Finally, the oocytes were expeditiously placed onto glass slides and examined using confocal microscopes. ImageJ V1.8.0 software was used to calculate the ratio of red to green fluorescence, which represented the MMP level of the oocytes.

### 2.9. Real-Time Quantitative PCR Analysis

A SteadyPure Universal RNA Extraction Kit (ACCURATE BIOLOGY, Cat# AG21022) was used to extract the total RNA 30~50 COCs or oocytes in each group. cDNA was synthetized using an Evo M-MLV RT Mix Kit from the same manufacturer. The primers of different genes are exhibited in Table 1. The real-time PCR reaction system (10 µL) included the following: 2× SYBR Green Pro Taq HS 5 µL; forward primer 0.2 µL, reverse primer 0.2 µL; cDNA and ddH_2_O 4.6 µL. The real-time PCR was conducted with a fast real-time PCR system (ABI Step One Plus). The 2^−ΔΔCt^ method was used to analyze the genes’ relative expression.

### 2.10. Statistical Analysis

For each experimental project, a minimum of 30 COCs must be cultured in vitro and used for maturity rate statistics and no less than 20 for TZPs, spindle morphology, and mitochondrial function statistics. All experimental items were tested with three biological replicates, and the outcomes were presented as means ± SEM. The data were analyzed using a *t*-test through the GraphPad Prism 7.00 software (GraphPad, Boston, MA, USA), which was utilized to carry out all analyses. For image analysis, average fluorescence intensity was analyzed using ImageJ V1.8.0 software. The average fluorescence intensity formula is as follows: average fluorescence intensity = total fluorescence intensity of the area/the area. Statistical significance was determined by a *p*-value of less than 0.05 and indicated via different symbols, with *p* < 0.05 represented by *, *p* < 0.01 represented by **, *p* < 0.001 represented by ***, and *p* < 0.0001 represented by ****.

### 2.11. Experimental Design

Experiment 1: Effect of CNP pre-treatment on oocyte meiotic arrest and maturation.

COCs were cultured in a CNP pre-treatment medium for 6 h, and the Con group was cultured in IVM medium for 6 h. Then, the cGMP and cAMP content in oocytes were measured and evaluated the nuclear stage. COCs were cultured in a CNP pre-treatment medium for 6 h, followed by 24 h IVM. The Con group was cultured in IVM medium for 24 h. Then, the oocyte maturation was evaluated.

Experiment 2: Effect of CNP pre-treatment on the TZPs of the oocytes.

COCs were cultured in a CNP pre-treatment medium for 6 h. The Con group was cultured in IVM medium for 6 h. Then, qRT-PCR and fluorescence staining were used to detect the related indicators.

Experiment 3: Effect of CNP pre-treatment on the spindle assembly of the oocytes.

COCs were cultured in a CNP pre-treatment medium for 6 h, followed by 14 h IVM. The Con group was cultured in IVM medium for 14 h. Then, qRT-PCR and fluorescence staining were used to detect the related indicators.

Experiment 4: Effect of CNP pre-treatment on the antioxidant capacity and mitochondrial function of the oocytes.

The COCs were cultured in a CNP pre-treatment medium for 6 h, followed by 24 h IVM. The Con group was cultured in IVM medium for 24 h. Then, qRT-PCR and fluorescence staining were used to detect the related indicators of mature oocytes.

## 3. Results

### 3.1. CNP Treatment Maintained Meiotic Arrest and Improved Oocyte Maturation

**Figure 1:**
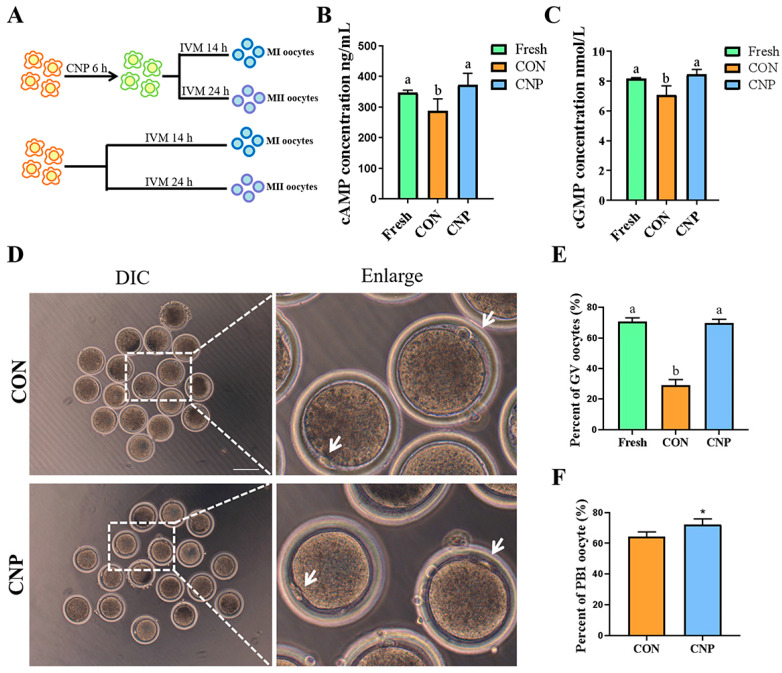
CNP treatment improved oocyte maturation in vitro. (**A**) The timeline diagram of experimental design. (**B**) cGMP concentration in goat oocytes. Different letters indicated significant differences. (**C**) cAMP concentration in goat oocytes. Different letters indicate significant differences. (**D**) DIC images of the oocyte PB1 in the CON and CNP treatment groups. White arrows indicate PB1. Bar = 100 μm. (**E**) Effect of CNP on goat oocytes’ meiotic arrest. (**F**) Rate of polar body extrusion of the CON and CNP treatment groups. *, *p* < 0.05.

We first evaluated the effect of 50 nM CNP treatment for 6 h on goat oocyte meiotic arrest. The results indicated that 50 nM CNP treatment for 6 h significantly maintained the oocytes’ cGMP and cAMP concentration compared with the CON group and was similar to the Fresh group (cGMP: Fresh group vs. CON group vs. CNP group: 8.16 ± 0.08 vs. 7.09 ± 0.60 vs. 8.46 ± 0.32; different letters, *p* < 0.05, Figure 1A; cAMP: Fresh group vs. CON group vs. CNP group: 347.7 ± 7.09 vs. 288.34 ± 38.74 vs. 372.57 ± 37.67; different letters, *p* < 0.05, Figure 1B). After CNP treatment, the oocytes were able to be maintained at the GV stage (Fresh group vs. CON group vs. CNP group: 70.63 ± 2.63 vs. 28.95 ± 3.89 vs. 69.99 ± 2.24; different letters, *p* < 0.05; Figure 1C,D). Next, we observed that CNP treatment increased PB1 compared to the CON group (CON group vs. CNP group: 64.25 ± 2.97 vs. 72.37 ± 3.44, *p* < 0.05; Figure 1E). Overall, these results indicated that CNP treatment before IVM maintained the goat oocyte meiotic arrest and improved oocyte maturation.

### 3.2. CNP Treatment before IVM Maintained TZPs of COCs

**Figure 2:**
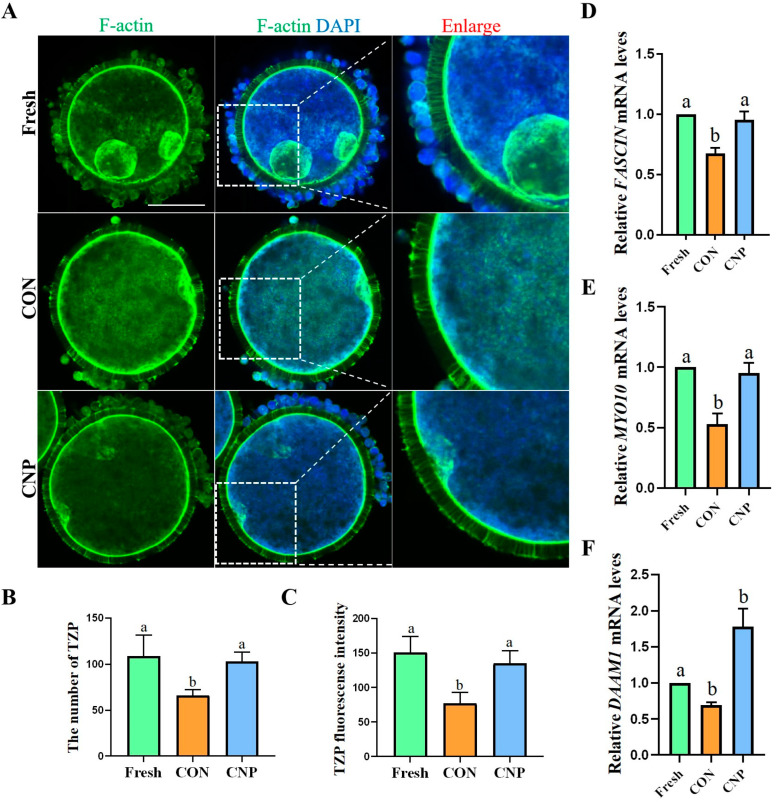
Effect of CNP on the TZPs of COCs. (**A**) Representative image of TZPs in Fresh, CON, and CNP treatment COCs. F-actin (Green). Bar = 50 μm. (**B**) The number of TZPs in Fresh, CON, and CNP treatment COCs. Different letters indicated significant differences, *p* < 0.05. (**C**) The fluorescence intensity of the F-actin signals was quantified in the Fresh, CON, and CNP treatment COCs. Different letters indicated significant differences, *p* < 0.05. (**D**–**F**) Expression of TZP assembly-related genes was detected. Different letters indicated significant differences, *p* < 0.05.

During IVM, TZP loss occurs uniformly 4–8 h later after the oocyte separates from the follicle and the oocyte resumes meiosis [2]. We examined whether the TZPs were maintained in the COCs after CNP treatment for 6 h. The results showed that the TZPs decreased rapidly in the CON group, whereas the CNP-treated COCs displayed an obviously higher TZP density that was similar to that of the Fresh COCs (Figure 2A). The TZPs’ quantitative results showed that the number of TZPs in the Fresh and CNP groups was notably higher than that in the CON group (Fresh group vs. CON group vs. CNP group: 108.67 ± 23.03 vs. 66 ± 6.24 vs. 103 ± 10.15; different letters, *p* < 0.05; Figure 2B). The fluorescence intensity analysis was consistent with this (Fresh group vs. CON group vs. CNP group: 150.93 ± 22.75 vs. 77.06 ± 16.00 vs. 135.03 ± 18.36; different letters, *p* < 0.05; Figure 2C). Additionally, the mRNA expression of the TZP assembly-related genes was measured. The data showed that the *FASCIN*, *MYO10*, and *DAAM1* mRNA expression notably increased after CNP treatment (*p* < 0.05, Figure 2D–F). These data indicate that CNP maintains cumulus–oocyte communication by influencing TZP assembly gene expression.

### 3.3. CNP Treatment before IVM Improved Spindle Assembly in Oocytes

**Figure 3:**
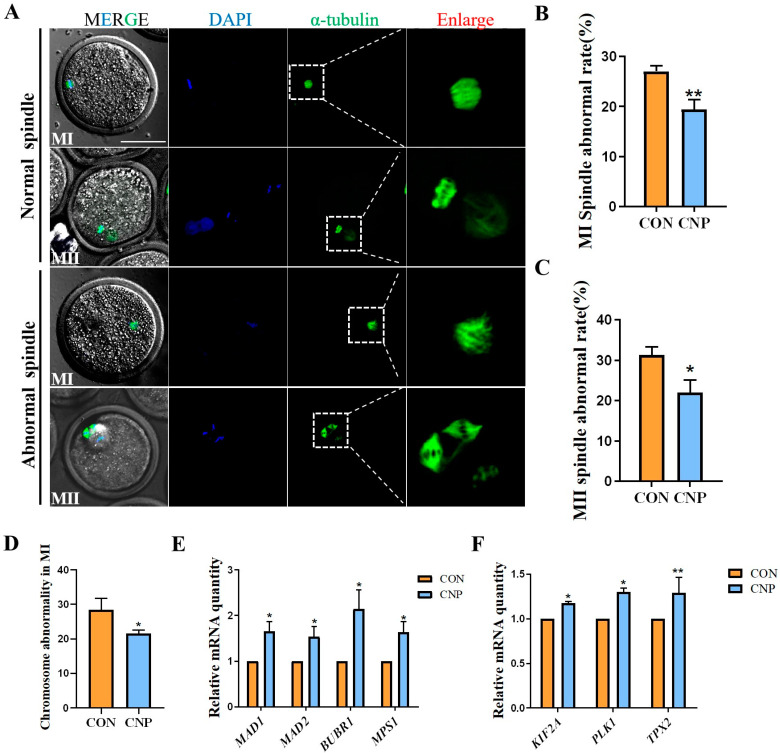
Effect of CNP on spindle assembly in goat oocytes. (**A**) Typical image of normal and aberrant spindles during meiosis. (**B**) Oocytes’ proportion with MI abnormal spindle in the CON and CNP treatment groups. **, *p <* 0.01. (**C**) Proportion of oocytes with MII abnormal spindle in the CON and CNP treatment groups. *, *p <* 0.05. (**D**) Proportion of MI oocytes with a misalignment chromosome in the CON and CNP treatment groups. (**E**) Expression of spindle assembly checkpoint-related genes were detected. *, *p <* 0.05. (**F**) SAC-related gene expression was detected. *, *p <* 0.05; **, *p <* 0.01.

An improvement in CNP during oocyte maturation and blastocyst development has been previously reported in several species. Hence, we explored whether CNP treatment could improve spindle assembly during meiosis. Normal and aberrant spindle images are shown in Figure 3A. The data showed that CNP significantly suppressed the aberrant spindle rate of MI-stage oocytes (CON group vs. CNP group: 27.06 ± 1.11% vs. 19.44 ± 1.88%; *p* < 0.01; Figure 3B) and MII-stage oocytes (CON group vs. CNP group: 31.33 ± 1.96 vs. 22.04 ± 3.09, *p* < 0.05; Figure 3C). Meanwhile, CNP treatment reduced chromosome misalignment in MI-stage oocytes (CON group vs. CNP group: 28.43 ± 3.35 vs. 21.5 ± 1.04, *p* < 0.05; Figure 3D). The mRNA expression of spindle assembly-related genes, *KIF2A*, *PLK1*, and *TPX2*, as well as the SAC-related genes *MPS1*, *MAD1*, *MAD2*, and *BUBR1,* were significantly increased in the CNP group (Figure 3E,F). Overall, these results showed that CNP treatment improved spindle assembly during oocyte meiosis.

### 3.4. CNP Treatment before IVM Enhanced the Antioxidant Capacity of Oocytes

**Figure 4:**
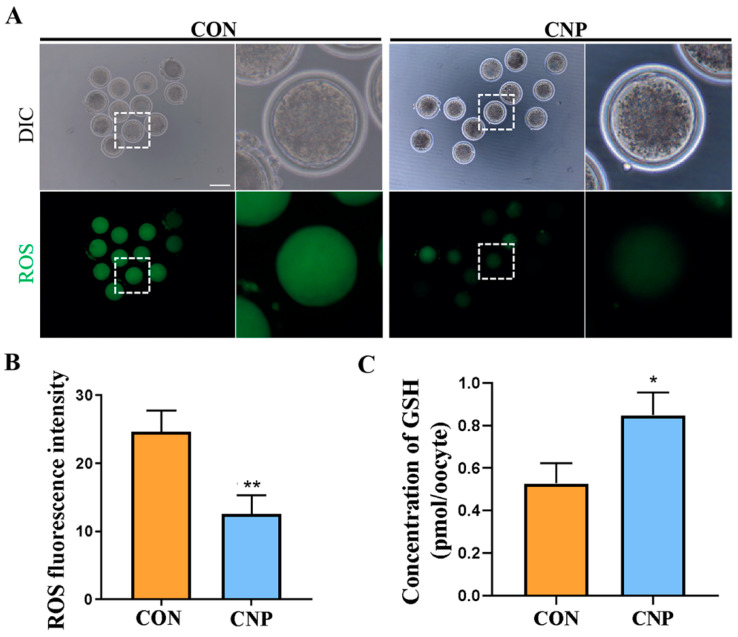
Effects of CNP on the ROS content and GSH levels in goat oocytes. (**A**) Images of ROS contents reflected by DCFH staining in the CON and CNP groups. Bar = 100 μm. (**B**) The ROS signal fluorescence intensities were quantified in the CON and CNP groups. **, *p <* 0.01. (**C**) The GSH levels were measured in the CON and CNP groups. *, *p <* 0.05.

Oocyte oxidative stress inevitably occurred during in vitro maturation and was more likely to occur than during in vivo maturation. Antioxidant capacity was one of the indicators of oocyte quality. Therefore, according to the transcriptome data, we examined the effect of CNP on the oxidative stress of MII oocytes. As shown in Figure 4A, the ROS levels were lower in the CNP treatment group. This was also supported via the fluorescence intensity analysis (CON group vs. CNP group: 24.64 ± 3.11 vs. 12.58 ± 2.70 *p* < 0.01; Figure 4B) and higher GSH levels (CON group vs. CNP group: 0.53 ± 0.09, *n* = 90 vs. 0.85 ± 0.11 *n* = 93, *p* < 0.05; Figure 4C).

### 3.5. CNP Treatment before IVM Improved Oocytes’ Mitochondrial Membrane Potential and Mitochondria Distribution

**Figure 5:**
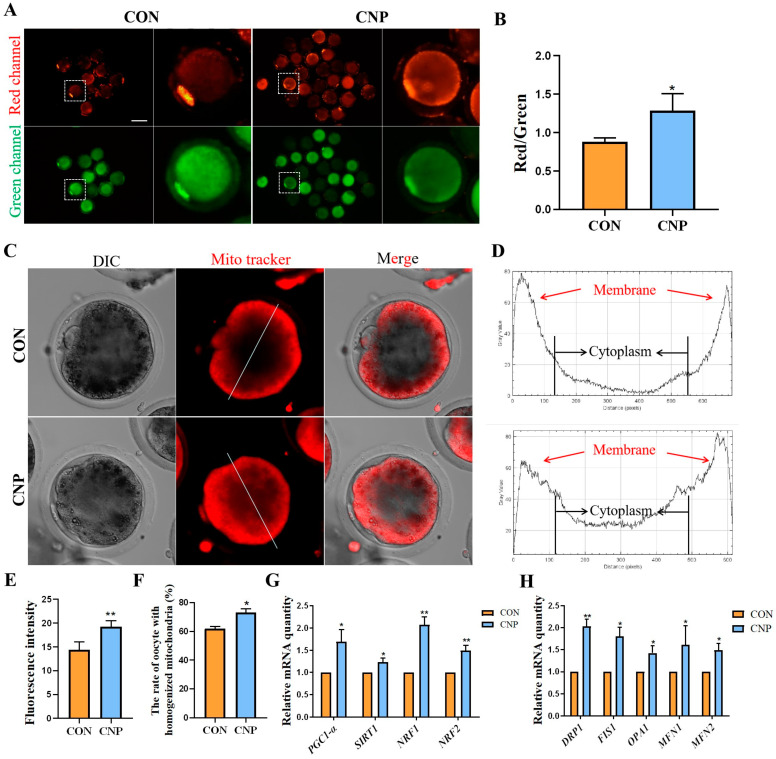
Effects of CNP on the mitochondrial distribution and function in goat oocytes. (**A**) Images of the green and red channels of JC1 staining in the CON and CNP groups. Bar = 100 µm. (**B**) The red/green ratio of the JC1 signals in the CON and CNP groups. *, *p* < 0.05. (**C**) Typical images of mitochondrial distribution in the CON and CNP groups. Bar = 50 μm. (**D**) The graphs showed the fluorescence intensity profiling of mitochondrial distribution in the CON and CNP treatment group oocytes. Pixel intensities were measured along the lines drawn across the oocytes. (**E**) Mitochondrial fluorescent intensity analysis in the CON and CNP groups. **, *p* < 0.01. (**F**) The proportion of oocytes with homogenized mitochondria in the CON and CNP treatment groups. *, *p* < 0.05. (**G**) Mitochondrial oxidative metabolism-related gene mRNA were detected. *, *p <* 0.05; **, *p* < 0.01. (**H**) Mitochondria fusion- and fission-related gene mRNA were detected. *, *p* < 0.05; **, *p* < 0.01.

Mitochondria distribution and activity are essential for oocyte maturation, fertilization, and embryonic development [6]. First, we evaluated MII oocytes’ mitochondrial membrane potential (MMP) using JC-1 staining. In the CNP treatment group, the MMP was notably higher (Figure 5A). The fluorescence intensity of red/green also showed a consistent trend (CON group vs. CNP group: 0.88 ± 0.05 vs. 1.29 ± 0.22, *p* < 0.05; Figure 5B), with these results indicating a higher level of mitochondrial activity in the CNP treatment group. Next, we used MitoTracker Red staining to visualize the mitochondria distribution. As shown in Figure 5C,D in the oocytes from the CON group, the mitochondria were located surrounding the cortical regions, showing a peripheral distribution pattern. Conversely, in the CNP group, the mitochondria exhibited a more homogeneous distribution pattern in the cytoplasm. Furthermore, the analysis of fluorescence intensity found that the mitochondrial content in the CNP treatment group was greater than that in the CON group (CON group vs. CNP group: 14.433 ± 1.67 vs. 19.28 ± 1.26, *p* < 0.01; Figure 5E). Meanwhile, we found that the ratio of oocytes with homogenized mitochondria was significantly higher in the CNP treatment group compared to the CON group (CON group vs. CNP group: 61.9 ± 1.56% vs. 73.04 ± 2.73%, *p* < 0.01, Figure 5F). In addition, the expression of mitochondrial oxidative metabolism genes, including *PGC-1α*, *SIRT1*, *NRF1,* and *NRF2,* was significantly increased in the CNP treatment group (*p* < 0.05; Figure 5G). Additionally, mitochondria fusion and division-related genes—*DRP1*, *FIS1*, *OPA1*, *MFN1*, and *MFN2*—were also significantly up-regulated in the CNP treatment group (*p* < 0.05; Figure 5H). In summary, these data showed that a period of CNP treatment before IVM significantly improves the mitochondrial activity and distribution, as well as the oocytes’ antioxidant capacity.

A detailed description of the qRT-PCR results:

In Figure 2F, FASCIN: Fresh vs. CON vs. CNP: 1 vs. 0.67 ± 0.04 vs. 0.95 ± 0.07. In Figure 2G, MYO10: Fresh vs. CON vs. CNP: 1 vs. 0.53 ± 0.08 vs. 0.95 ± 0.08. In Figure 2H, DAAM1: Fresh vs. CON vs. CNP: 1 vs. 0.69 ± 0.04 vs. 1.78 ± 0.25. In Figure 3E, MAD1: CON vs. CNP: 1 vs. 1.65 ± 0.20. MAD2: CON vs. CNP: 1 vs. 1.53 ± 0.22. BUBR1: CON vs. CNP: 1 vs. 2.143 ± 0.42. MPS1: CON vs. CNP: 1 vs. 1.64 ± 0.23. In Figure 3F, KIF2A: CON vs. CNP: 1 vs. 1.17 ± 0.02. PLK1: CON vs. CNP: 1 vs. 1.30 ± 0.05. TPX2: CON vs. CNP: 1 vs. 1.29 ± 0.17. In Figure 5G, PGC1-α: CON vs. CNP: 1 vs. 1.69 ± 0.27. SIRT1: CON vs. CNP: 1 vs. 1.23 ± 0.09. For the relative NRF1 mRNA leves were CON vs. CNP: 1 vs. 2.07 ± 0.18. NRF2: CON vs. CNP: 1 vs. 1.50 ± 0.12. In Figure 5G, DRP1: CON vs. CNP: 1 vs. 2.04 ± 0.16. FIS1: CON vs. CNP: 1 vs. 1.80 ± 0.20. OPA1: CON vs. CNP: 1 vs. 1.43 ± 0.16. MFN1:CON vs. CNP: 1 vs. 1.62 ± 0.43. MFN2: CON vs. CNP: 1 vs. 1.49 ± 0.16.

## 4. Discussion

The present study aimed to investigate the effect of CNP administration on goat oocyte maturation, with a focus on the underlying molecular pathways. Our findings indicated that CNP played a crucial role in enhancing cytoskeleton dynamics, particularly in terms of facilitating the assembly of TZPs and organizing the spindle. Additionally, CNP treatment exerted a significant influence on mitochondrial activity and the maintenance of oocyte redox homeostasis.

CNP is a peptide consisting of 22 amino acids that are widely distributed in the central nervous system, endothelium, and gastrointestinal and genitourinary tracts [19,20]. In recent years, the effects of CNP on mammalian folliculogenesis, oocytes, and embryonic development have been reported [6,21,22]. Thus, we delved into the mechanisms by which CNP improved the developmental potential of goat oocytes. First, we examined the changes in cGMP and cAMP, the downstream signals of CNP, and the effect these changes had on meiosis arrest and PB1. These results are similar to those in previous studies that have described the CNP’s effect on oocytes from other species [3,23,24]. Based on the above results, we posit that the functions of CNP might be involved in biological processes during goat oocyte meiosis.

As an essential component of the communication bridge between granulosa cells and oocytes, TZPs are known to participate in the growth and development of mammalian oocytes and the regulation of meiosis [25]. Thus, we then tried to explore whether CNP is involved in the regulation of the communication between cumulus cells and oocytes. Our data indicated that CNP treatment blocked the shrinkage of TZPs, and the block might be caused by the elevated cGMP induced by CNP. These results were similar to those of previous studies that show that cGMP stabilizes TZPs through the ERK-MAPK pathway in mouse COCs [26]. TZPs are specialized filopodia that are composed primarily of an actin core and a small amount of tubulin. The actin core typically comprises DAAM1, FASCIN, and MYO10 [27]. In line with this, we found that CNP maintained the expression of these genes. In summary, CNP is essential for the communication between granulosa cells and oocytes.

The quality of mature oocytes plays a crucial role in determining fertilization and embryonic development. The process of oocyte maturation necessitates the concomitant maturation of both the nucleus and cytoplasm. The precise spindle assembly and segregation of chromosomes during nuclear maturation are dependent on microtubules, thereby promoting a decrease in aneuploidy [28]. In our previous study, we found that CNP enhances immature mouse oocyte maturation and developmental competence by improving spindle morphology and chromosome alignment [27]. In this study, similar results confirmed that CNP was able to reduce the appearance of abnormal spindles and inaccurate chromosome alignment in goat oocytes in vitro. Intracellular monitoring mechanisms play a vital role in ensuring the accurate separation of chromosomes, with the spindle assembly checkpoint (SAC) serving as an important mechanism to maintain the fidelity of chromosome separation [28]. During the process of microtubule–kinetochore attachment, unstable attachment leads to the activation of SAC. SAC-related proteins mainly include the Bub and Mad protein families, including Mad1, Mad2, Mad3/BubR1, Bub1, Bub3, and Mps1. When a cell divides, these proteins are recruited to the centromere to monitor the arrangement of chromosomes on the equatorial plate and ensure the proper separation of chromosomes [29]. Bub1 is initially localized at the centromere and recruits other SAC proteins. Bub3 and BubR1 are required to monitor the cell cycle and ensure the accuracy of chromosome segregation [30]. The findings of a study revealed that melatonin serves as a facilitator in the development of vitrification of mouse oocytes by means of the regulation of SAC-related genes [31]. Hence, we undertook an assessment of the expression of those genes, and our results showed that CNP treatment resulted in an increase in the expression of SAC-related genes. The improved alignment of chromosomes in the CNP treatment group may be accounted for by the explanation provided.

The oocyte in an in vitro culture environment usually adopts a gas phase environment of 5% CO_2_ and 95% air (20% O_2_), and its partial oxygen pressure is much higher than that in vivo. Therefore, oocytes in vitro are often exposed to oxidative stress due to the high oxygen partial pressure [32]. With this in mind, we first tested the ROS and GSH levels in the different treatment groups as they both reflect the oocyte’s redox status. According to our results, CNP significantly increased the antioxidant capacity of the oocytes, which is similar to the findings of other studies [33,34]. The mitochondrion respiratory chain synthesizes the ATP through an oxidative phosphorylation system for many biological processes during oocyte maturation. After oocyte maturation, mitochondria migrate to the central region of the oocyte, and the homogeneous distribution of mitochondria is considered a sign of oocyte cytoplasmic maturation [35]. The maintenance of mitochondrial integrity and homeostasis is achieved through the processes of fusion and fission. Any disruption to these processes may result in an accumulation of ROS that exceeds the capacity of the scavenging mechanisms, leading to oxidative stress and, ultimately, apoptosis in oocytes [36]. Therefore, the number and activity of mitochondria are critical criteria for evaluating oocyte quality. Our results showed that CNP treatment improved the functions of mitochondria in goat oocytes, evidenced by the homogenization of mitochondrial distribution, an increase in mitochondrial membrane potential and oxidative metabolism, and an increase in the expression of genes related to fusion and division. This may lead to a sufficient supply of ATP required for oocyte development, thus resulting in higher-quality oocyte development.

## 5. Conclusions

In conclusion, our research has established the importance of CNP in regulating oocyte maturation in goats. Specifically, CNP facilitates intercellular communication between somatic cells and oocytes, promotes spindle assembly, and enhances mitochondrial function during oocyte IVM. These findings offer valuable insights into the physiological mechanisms underlying the CNP-mediated regulation of oocytes. Furthermore, this study carries significant implications for improving in vitro oocyte culture systems, which can generate a greater number of high-quality oocytes for auxiliary breeding technology and enhance the economic benefits of animal husbandry.

## Figures and Tables

**Table 1:** Primers used for quantitative reverse-transcription PCR.

Gene Classification	Gene	Primer Seq (5′-3′)	Fragment Size (bp)	Annealing Temperature
Reference gene	*ACTB*	F: CGCAGACAGGATGCAGAAAG	148	60 °C
		R: GCTGATCCACATCTGCTGGA		
TZP assembly-related genes	*DAAM1*	F: TGATGTCACCTGCGAGTTGG	78	60 °C
		R: CGCTCCGGGTACAATGGAAT		
	*FSCN1*	F: AAGTACTGGACGCTGACGAC	133	
		R: CCGTCACGAACTTGCCATTG		
	*MYO10*	F: TGGAGTCGCTCAACTTCGAC	118	
		R: TTGAAGCTAGGCTTCTCCGC		
Spindle assembly-related genes	*KIF2A*	F: GCACAACAGAATGCACGTAG	132	60 °C
		R: GTTTGTAGCATCAACATCCTGGG		
	*PLK1*	F: GATGCTTCAGCCAGATCCCA	200	
		R: CGGGGTTCTCCATGCCTTTAT		
	*TPX2*	F: GGAGAGGTGCCCAAGTTCAA	283	
		R:GCTGAAAAGGTTCCTGAACGATAA		
SAC-related genes	*MAD1*	F: TGGAGGGAGGATCTGGACTG	226	60 °C
		R: ATCTCGCGCTCGTAGTTCCT		
	*MAD2*	F: TGCTTTTGAAACGAGTGGCG	175	
		R: ACCAAATGAGAAGAACTCGGC		
	*BUBR1*	F: TCCCAGCACAGACAATTCCA	168	
		R: CCCTACACGGCTGATTGGAG		
	*MPS1*	F: CGGCAGATTCCAGAGCAGAA	140	
		R: TCATCCAAGGCACTGTTGCT		
Mitochondrial oxidative metabolism-related genes	*PGC-1α*	F: AAGCCAACCAAGATAACC	157	60 °C
		R: TACAACTCAGACTGCTCGGG		
	*SIRT1*	F: GGCTTACAGGGCCTATCCAG	124	
		R: CACCAAACAGAAGGTTATCTCGG		
	*NRF1*	F: AGGCTGGGGCAAAGAAAG	303	
		R: CCAACCTGGATAAGCGAGAC		
	*NRF2*	F: CCAACTACTCCCAGGTAGCCC	227	
		R: AGCAGTGGCAACCTGAACG		
Mitochondria fusion and division-related genes	*DRP1*	F: GAGAAGAAAATGGAGTTGAAGCA	232	60 °C
		R: CACCTACAGGCACCTTGGTC		
	*FIS1*	F: GGAACTACCGGCTCAAGGAAT	206	
		R: GGACACAGCAAGTCCGATGA		
	*OPA1*	F: AGCTTCTGACCTACTTCTCTTGT	110	
		R: TCTCTTTGTCTGACACCTTTCTGT		
	*MFN1*	F: CGGGCACAGATGTCACTA	151	
		R: GGCTTGGAAAGTCGCTCA		
	*MFN2*	F: TGCATGAAACTGGCGCGAT	282	
		R: TGATGCCCCTCACTTTGGAC		

## Data Availability

All data generated or analyzed during this study are included in this published article.
